# Community-Based Measures to Against the COVID-19: An Experience From Vietnam With Consecutive 3 Months of no New Infection in the Community During the First Wave of Pandemic

**DOI:** 10.3389/fpubh.2021.583655

**Published:** 2021-07-21

**Authors:** Nguyen Hai Nam, Bao-Tran Do Le, Nguyen Tien Huy

**Affiliations:** ^1^Graduate School of Medicine, Kyoto University, Kyoto, Japan; ^2^Faculty of Medicine, University of Medicine and Pharmacy at Ho Chi Minh City, Ho Chi Minh City, Vietnam; ^3^Department of Biochemistry, University of California, Los Angeles, Los Angeles, CA, United States; ^4^School of Tropical Medicine and Global Health, Nagasaki University, Nagasaki, Japan

**Keywords:** outbreak, control measures, epidemiological F0–F5 tracing system, Vietnam, COVID-19

## Abstract

Vietnam has faced a high risk of transmission of COVID-19 during the pandemic. Despite the specific challenges that come with a low-resource country, the Vietnamese government has provided a sustainable response, demonstrating both great capacity and rapid decision-making to manage the entirety of the COVID-19 outbreak with lessons learned from the SARS and H1N1 epidemics in 2003 and 2009, respectively. A rapid response, specific epidemiological F0–F5 tracing system, and public education are some of the key measures that have helped Vietnam to control the outbreak. As of July 15, 2020, Vietnam has reported 373 accumulated confirmed cases and no deaths within the last 90 consecutive days of no new infections in the community. Vietnam can now consider declaring an end to the COVID-19 crisis on their part.

## Introduction

The outbreak of COVID-19 spread rapidly during the period of the traditional Lunar New Year Festival in Vietnam, China, and Taiwan; the occasion saw a stream of millions of people who were expected to visit their home countries (1). Given the high risk of virus transmission since the first case report of COVID-19 in China on December 1, 2019 (2), and the first case outside China in Thailand on January 13, 2020 (3), the Vietnamese government took numerous preventive strategies. Indeed, airports and hospitals established additional stations to deploy body temperature scanning for passengers and patients who had entered Vietnam in the last 21 days. Early detection and high priority surveillance of infected cases as well as strict monitoring of airports, seaports, and national borders were seriously applied. On January 30, 2020, when the World Health Organization declared COVID-19 a Public Health Emergency of International Concern with 7.736 confirmed cases in China and 82 confirmed cases in 18 other countries (4), Vietnam had stopped issuing visas for Chinese citizens and foreign travelers who visited China in the past 14 days. All Vietnamese citizens had been urged to complete their health status declarations via Bluezone, a bluetooth-based mobile application that helps to notify its users if they come into contact with a COVID-19 patient (5). All passengers who entered Vietnam from affected areas must report all information, including their contact details, COVID-19 symptoms, and current and previous locations at entry ports before being sent to temporary hospitals for 14-day self-isolation management. The field hospitals were built from scratch and were also launched in the suburbs.

## How the Vietnamese Government Managed the First Wave of the Pandemic

The Vietnamese Centers for Disease Control and Prevention, which had been developed and improved since the outbreak of SARS in 2003 and H1N1 in 2009, cooperating with the Ministry of Health, had promptly advised the government to achieve effective and rapid control of various items. These included (1) social distancing and barriers, restriction of movement, avoiding public transport, closure of schools and public areas, prohibition of gatherings of more than 15 people in air-tight areas, obligatory use of face masks and hand hygiene practice, and enhanced environmental ventilation. In addition, advice also looked at (2) preventative measures, such as quarantining suspicious cases, isolating confirmed cases, performing epidemiological tracing, and contacting people who are related to a confirmed case of COVID-19 by using the F0 to F5 evaluation system (Table 1). Since February 14, Vietnamese citizens coming home from abroad and foreign citizens arriving in Vietnam must be quarantined for 14 days and tested for COVID-19. Further issues included (3) advice on socio-economic management was also given: adjustment of relevant health insurance policies, tax, the workforce, and food and daily necessities; (4) mobilization of armed forces to control the air, sea, and land borders; and (5) social media education to fight fake news and the spread of misinformation.

**Table 1:** F0–F5 evaluation system for epidemiological tracing contacting people who are related to a confirmed case of COVID-19 in Vietnam.

**Level**	**Definition and the attitutde of tracking**
F0	Positive with COVID-19 (isolation and treat in the hospital)
F1	Suspected of COVID-19 or having close contact with F0 (should wear mask, maintain a distance to other people for at least 2 m, should go to the hospital for isolation, inform to the Government and F2 about their situation
F2	Having close contact with F1 (should wear mask, maintain a distance to other people for at least 2 m, should stay at home for isolation and inform to the Government and F3 about their situation)
F3	Having close contact with F2 (should wear mask, should stay at home for isolation, and inform to the Government and F4 about their situation)
F4, F5	Having close contact with F3, F4 (should wear mask, should stay at home for isolation, and inform to the Government)

**Figure 1:**
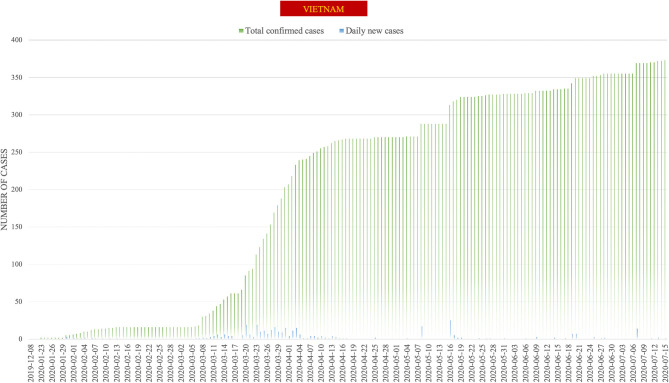
Epidemic curve of new cases of COVID-19 in Vietnam. The total confirmed cases are presented in green and the daily new cases in blue. The using data was retrieved from the daily reports released by the World Health Organization.

On March 19, the Vietnamese government temporarily achieved a triumph for the first stage of this pandemic with 85 confirmed cases and no cases of death since the first Vietnamese case of COVID-19 was acquired from China on January 23, 2020 (6, 7). Vietnam also had a period of 2 weeks that was free of new cases before having a series of confirmed and suspected cases from March 6 onwards after a stream of Vietnamese people returning from overseas (8). Subsequently, Vietnam has entered a new period characterized by susceptibility to COVID-19 infection due to the high number of clusters in the community and the loss of source tracing. On March 20, the Government decided to impose a lockdown on Bach Mai hospital, which is the biggest national healthcare center in the North of Vietnam and which sees a high volume of outpatients, since the two female nurses were tested positively to COVID-19 without a detectable source. The Prime Minister was quick to implement a lockdown level of 3 and 4 that saw the strict application of contact investigation strategy using the F0–F5 evaluation system. All of Bach Mai Hospital, including its 7,664 staff members, was quarantined following positive testing results for SARS-CoV-2 in 27 employees working in catering (9). Contact tracing in the community was carefully and quickly processed in an additional 52,239 individuals. Among them, 27,893 F1 and F2 persons were subsequently detected and placed under quarantine using ~30,000 RT-PCR tests. The national lockdown requested that citizens stay at home and just go out for daily necessities, such as food shops, pharmacies, urgent medical appointments, and work. School and universities were closed. Despite the school closures, education systems moved to online activities as an alternative. Online teaching and learning platforms were encouraged and widely used. Most businesses began shutting down except those that were essential. Suspension of public transportation followed, and travel between cities was extremely limited. A total of 3 week later, the Bach Mai Hospital crisis was consequently contained without further spread. As of July 15, 2020, our report revealed 373 confirmed cases of COVID-19, with 352 patients (94.4%) having recovered from COVID-19, and no cases of death (10). Vietnam had 90 consecutive days of being free from new acquired COVID-19 cases in the communities (Figure 1). At present, the Vietnamese government remains vigilant of the infectious virus to cope with its next wave. The AstraZeneca vaccine had been approved to use across the country. The same tactics coupled with a serious attitude are maintained.

## Key Weapons During a Time of Crisis: Rapid Response, a Specific Epidemiological F0–F5 Tracing System, and Public Education

The Vietnamese Government quickly ramped up its responses and prepared for combat following the first case that originated in China (7). A national declaration of an epidemic was officially announced on February 1, 2020, while there were still only six confirmed cases. The prompt reaction at the begining of February remains a key point of success, as demonstrated in other countries. In Romania, rapid implementation of containment measures, including the declaration of a state of emergency and swift application of 14-day self-isolation, coupled with the establishment of a nationwide information campaign dramatically lessened the surge of infectious cases (11). In the same manner, it took China 10 days to construct 45 makeshift hospitals in response to the burden of new confirmed cases. Early reactions from the government in association with sufficient emergency responses significantly contributed to the reopening of Wuhan, the initial epicenter of COVID-19, just after 76 days of strict restrictions (12). Furthermore, aggressive prevention with epidemiological tracing using the F0–F5 evaluation system represent an unique but effective measure to mitigate the crisis. Regarding financial assisstance, given the limited average budget of households for healthcare services as well as the insufficient coverage of the insurance, the Vietnamese government had anticipated an overwhelming burden of medical expenditures on a large scale during the outbreak. Since Vietnamese healthcare consumers are unable to afford healthcare payments equal to or higher than US$90 (VND 2 million), especially in terms of uninsured, non-married, and unemployed individuals (13), the government offered budget support packages of US$2.6 billion in which a sum of US$80 (VND 1.8 million) was distributed monthly between April and June (14). Another important implication is the consistent coordination across sectoral ministries/the government departments in dealing with an event. Right after the notice of suspected patients who returned to Vietnam by airlines, the Ministry of Transport rapidly alerted the relevant agencies about the passengers' information and the corresponding flight numbers so that quarantine could be implemented quickly and efficiently. Passengers were also given instructions from airlines regarding the current regulations on disease prevention and control in Vietnam. In parallel, the Ministry of National Defense took responsibility for the establishment of the quarantine areas and transporting all relevant people from or passing through regions hit by the epidemic and into other areas. Recognizing the risks of over-reaching the airports' capacities, the Ministry of Public Security had quickly promulgated necessary regulations to shorten the duration of immigration procedures. Side by side, the Ministry of Health and Foreign Affairs closely updated the epidemic reports from around the world and hourly reported to the Prime Minister any adequate and important findings.

On the other hand, the Government, with regular announcements on daily news of the crisis and up-to-date developments in terms of COVID-19 treatments, provided official and exact information to the public. The introduction of the website http://ncov.moh.gov.vn and the application of the NCOVI and Vietnam Health apps on mobile phones (15), which provided full information regarding preventive measures, new cluster detection, testing data, and live consultation for any inquiries related to the outbreak, sufficiently responded to the demand of citizens about the reliable sources of information. Furthermore, the dissemination of necessary information was universally enhanced by the official press and social media. The publication of ~15,000 articles from 14 online newspapers from January 9 to April 4, coupled with the hourly COVID-19 update in the two main social media channels, Facebook (more than 57% of the population), and the local app Zalo (with 100 million users), remarkably improved the awareness of all citizens about the infectious disease (16). Besides, a Vietnamese scientist had also engaged in the global research community by publishing and sharing data related to COVID-19. High-quality publications attracted attention from numerous readers and also significantly contributed to the global database and knowledge (7, 17, 18). As a consequence, all Vietnamese people felt safe, proud, and confident in the Government. The whole country is courageous and united in the efforts to combat COVID-19. Therefore, maintaining a high level of consciousness and responsibility for citizens is crucial and effective during this critical period, as previously reported in Jordan and South Korea (19, 20).

## Is it the Right Way, Vietnam?

Lessons learned from responding to the Ebola outbreaks in Africa (2014–2016 and 2018–2020) emphasize the crucial role of community engagement as well as rapid response in fighting against the crisis (21). Early detection of a new infection, comprehensive contact tracing, and strict quarantine of confirmed cases are some of the backbone policies of success in African countries. Vietnam, with the unique tracing system F0–F5 classification, has also proven its effectiveness in rapidly locating the new infected cluster and therefore maximally limit the spread of the virus. Furthermore, Vietnamese citizens seem in favor of adopting lockdown strategies and be in line with the full response of the authorities. Regarding non-pharmaceutical intervention, the implementation of compulsory quarantine, school closure, and bans of public gatherings in New York City during the 1918–1919 influenza pandemic reduced the total mortality and induced greater delays in reaching peak mortality (22). These physical and lockdown restrictions, such as the closure of borders and cities, non-essential businesses, and educational facilities, social distancing and barriers, and the shift to work from home also helped to flatten the new infection curve in Australia (23). Similarly, we also witnessed the rapid application of social distancing and barriers as well as epidemiologically preventive measures in Vietnam since the first day of the outbreak. The current situation of a consecutive 3 months without a new community infection might be the best answer for the aforementioned question.

## Conclusion

At the beginning of the pandemic, the strong public support for the response measures and a strong culture of surveillance were key points in the struggle for victory. Using strict and cross-sectoral control measures combined with social and economic support measures, the Vietnamese government gained the support of all Vietnamese citizens during the first wave of the pandemic. Various factors were involved: the evacuation flight to rescue 30 Vietnamese citizens in Wuhan, China; the free treatment and laboratory investigation of all confirmed and suspected COVID-19 cases; the warm attitude of hospital staff, high quality of surveillance, and appropriate daily regimen for quarantined people at temporary medical camps; and the stabilization of food and daily necessities. These all contributed to building the citizens' belief in the government. Following the epidemics of SARS in 2003 and H1N1 in 2009, coupled with strong support from the United States Center for Disease Control and Prevention (US CDC) and the WHO, the Vietnamese government have provided a sustainable response and executed a rapid response to manage the entirety of the COVID-19 outbreak. A serious control policies balancing with humanity in a well-organized and well-trained teams had responded adequately to the inquiries of Vietnam during this time. Vietnam can now consider declaring an end to the COVID-19 crisis.

## Data Availability Statement

The original contributions presented in the study are included in the article/supplementary material, further inquiries can be directed to the corresponding author/s.

## Author Contributions

NN: conceptualization, software, writing—original draft preparation, visualization, and investigation. B-TD: writing—original draft preparation, visualization, and investigation. NH: supervision, writing—reviewing, and editing. All authors contributed to the article and approved the submitted version.

## Conflict of Interest

The authors declare that the research was conducted in the absence of any commercial or financial relationships that could be construed as a potential conflict of interest.
